# Oral Vancomycin in the Treatment of *Clostridioides difficile* Infection: A Single-Center Observational Study in Southern Poland (2016–2022), Involving 528,887 Hospitalized Patients

**DOI:** 10.3390/antibiotics15020161

**Published:** 2026-02-03

**Authors:** Anna Pałka, Mateusz Gajda, Norbert Kapczyński, Estera Jachowicz-Matczak, Marcin Krzanowski, Jakub Kasprzyk, Barbara Żółtowska, Jacek Czepiel, Jadwiga Wójkowska-Mach

**Affiliations:** 1 Chair of Microbiology Medical Faculty, Jagiellonian University Medical College, 31-121 Kraków, Poland; anna.palka@szpitalmegrez.pl (A.P.); mateusz14.gajda@uj.edu.pl (M.G.); norbert.kapczynski@student.uj.edu.pl (N.K.); estera.jachowicz-matczak@uj.edu.pl (E.J.-M.); 2 Department of Nephrology and Transplantology, Jagiellonian University Medical College, 30-688 Kraków, Poland; marcin.krzanowski@uj.edu.pl; 3 University Hospital in Krakow, 30-688 Kraków, Poland; jkasprzyk@su.krakow.pl (J.K.); bzoltowska@su.krakow.pl (B.Ż.); 4 Department of Infectious Diseases, Jagiellonian University Medical College, 30-688 Kraków, Poland; jacek.czepiel@uj.edu.pl; 5 Faculty of Medicine and Health, University of Applied Sciences in Tarnow, 33-100 Tarnów, Poland

**Keywords:** *C. difficile*, hospital-acquired infection, CDI, antimicrobials, vancomycin, metronidazole

## Abstract

**Objectives:** *Clostridioides difficile* infection (CDI) remains a major healthcare challenge, particularly in resource-limited settings. **Methods:** This retrospective, single-center study analyzed CDI epidemiology and treatment outcomes among 528,887 hospitalized patients at the University Hospital in Kraków, Poland, between 2016 and 2022. **Results:** A total of 2341 CDI cases were confirmed, with an overall incidence of 4.32 per 1000 admissions. The highest rates were observed in geriatric and infectious diseases units. During the COVID-19 pandemic, healthcare-associated CDI cases surged, accounting for up to 89.2% of infections in 2020 with an incidence rate of 3.8 per 1000 admissions, compared with 2.5 per 1000 admissions in 2016. Vancomycin-based therapy was associated with significantly lower mortality (OR 0.73, 95% CI 0.56–0.95) compared to metronidazole, while combination therapy (vancomycin, metronidazole) showed the highest recurrence rate (17%). Fidaxomicin use was minimal (0.4%) due to limited availability. Recurrent CDI occurred in 14.2% of cases, with a relapse-free survival advantage observed in vancomycin-treated patients. The overall in-hospital case fatality rate associated with CDI was 22.5%. **Conclusions:** Despite stable overall CDI incidence, the study highlights the impact of increased antibiotic consumption during the pandemic on HA-CDI dynamics. The findings underscore the need for improved antimicrobial stewardship, broader access to advanced therapies such as fidaxomicin and bezlotoxumab, and enhanced diagnostic protocols. In settings with restricted therapeutic options, vancomycin remains a valuable treatment, particularly for reducing mortality.

## 1. Introduction

Infection of the colon with the Gram-positive bacilli *Clostridioides difficile* (*C. difficile*) can be potentially life-threatening, particularly in elderly individuals. The risk of *Clostridioides difficile* infection (CDI) is further increased in patients with gut microbiota dysbiosis, often resulting from prior antimicrobial drug exposure. CDI is the leading cause of health-care-associated infectious diarrhea [1].

The life cycle of *C. difficile* is influenced by antimicrobial agents, the host immune system, and the host microbiota and its associated metabolites. The primary mediators of inflammation in *C. difficile* infection (CDI) are large Clostridial toxins, toxin A (TcdA) and toxin B (TcdB), and, in some bacterial strains, the binary toxin CDT. The toxins trigger a complex cascade of host cellular responses to cause inflammation and tissue necrosis, leading to diarrhea—the major symptom of CDI [2]. However, transmission of *C. difficile* may occur in conditions unrelated to previous use of antibiotics [3].

*C. difficile* can manifest in a range of clinical syndromes ranging from asymptomatic colonization to CDI as inflammatory colitis characterized by diarrhea with abdominal pain and, in severe cases, death. CDI often leads to prolonged hospital stays, and increased case fatality rates, especially in elderly patients [4]. Vancomycin, fidaxomicin, and metronidazole are the current standard for CDI treatment; however, in addition to primary infection, one in five patients treated for CDI experiences recurrent disease. According to Feuerstadt et al.’s study in the medicare population, recurrent ICU occurs in up to 35% of patients aged ≥ 65 years and after each recurrent CDI episode, 20% of patients experienced a sepsis event [5]. Microbial strategies to combat severe colitis through fecal microbiota transmission (FMT) have seen great advancements in recent years. However, how FMT strategies alter mucosal immune defense and how the host immune system contributes to CDI pathogenesis are not fully understood. FMT is reserved for people with recurrent CDI who have failed two rounds of antibiotic treatment or have severe disease [6]. There is no agreed legal framework for Poland regarding FMT regulation [7].

CDIs are a significant cause of morbidity and mortality in the United States with an estimated 500,000 cases in the United States yearly [8]. The epidemiology data of CDI in Europe are more variable due to different reporting systems within the European Union. In European Union/European Economic Area (EU/EEA) countries in 2019 (the last of the reports before the COVID-19 pandemic), for healthcare-associated (HA) CDI, the reported mean hospital incidence density was 2.0 cases per 10,000 patient-days and for community-associated or unknown association (CA/UA) CDI, the reported mean hospital incidence was 0.7 cases per 1000 patient discharges or admissions [9]. However, by extrapolation of the data from the United Kingdom to Europe, they result in a total number of 172,000 CDI cases annually within the European Union [10]. The economic impact of CDI is enormous, leading to additional medical costs of over USD one billion per year in the United States and EUR three billion per year within the European Union [11]. Hospitalized patients and adults treated with antibiotics are especially at a higher risk of developing CDI [12].

Unfortunately, in Poland, there are no CDI surveillance—although there is an obligation to report cases to the national register—and antibiotic consumption (AMC) programs with infection prevention and control, although individual single-center studies indicate a major epidemiological problem with CDI and AMC. According to the general national data, in 2023, the incidence of CDI, both HA and CA/UA, was 2.7 per 1000 admissions [13], but the incidence in the population of patients undergoing hip or knee arthroplasty was higher, 3.4 per 1000 operations [14]. Additionally, in the above-described population, patients undergoing hip or knee arthroplasty who were prescribed at least one antibiotic in the post-operation period were diagnosed with CDI more often than patients who had no antibiotic treatment (55.0/1000 patients vs. 1.8/1000 patients) [14].

In the University Hospital in Krakow (UHK) in 2008–2014, the incidence rate was 2.87/1000 hospitalizations—and it was the expected level [15]—but, in 2019, antibiotic consumption was higher than expected, 23.9 DOT/100 pds in the non-ICU setting and 163.6 DOT/100 pds in the ICU. In the COVID-19 pandemic period, AMC was even higher—in non-COVID-19 patients, it was, respectively, 26.7 DOT/100 pds and 217.2 DOT/100 pds [16]. A very high prevalence of multidrug-resistant strains and horizontal transmission of *Acinetobacter baumannii* strains was also confirmed at that time in the UHK [17,18]. Therefore, our study aim was to investigate the epidemiological trends of CDI between 2016 and 2022, in the context of previously reported high levels of antibiotic consumption during the COVID-19 pandemic. The analysis focused particularly on the CDI treatment and the risk of recurrent CDI or death.

## 2. Results

Between 2016 and 2022, a total of 528,887 patients were hospitalized at the UHK. During this period, 16,156 tests for *Clostridioides difficile* infection (CDI) were ordered, representing 3.21% of all hospitalizations; ELISA confirmation was required in 14.4% of cases following an initial immunoenzymatic test (Table 1). Females comprised 52.1% of the tested population, and the median age of CDI-positive patients was 72.0 years.

A total of 2341 positive results were recorded, yielding a population incidence of 4.32 per 1000 patients (Table 1). An increase in overall CDI incidence was observed in the post-COVID-19 years, with rates of 5.05 and 4.62 per 1000 patients in 2021 and 2022, respectively (Table 2). Incidence rates varied significantly across subpopulations, with the highest rates observed in patients from the geriatric unit (23.9/1000) and the ID Unit (19.5/1000) (Table 3).

Overall, CA-CDI represented 24.8% of cases, with the lowest proportion in 2020, when HA-CDI predominated (89.2%) (Table 2). The incidence rate of HA-CDI was 3.24/1000 patients, while CA-CDI was 1.07/1000 patients. The HA-CDI/CA-CDI ratio varied and was from 2.0 in the pre-pandemic time to 6.7 in 2020, at the beginning of the COVID-19 pandemic. Next, in 2021–22, the ratio was slightly higher, 2.3 to 2.7.

**Figure 1:**
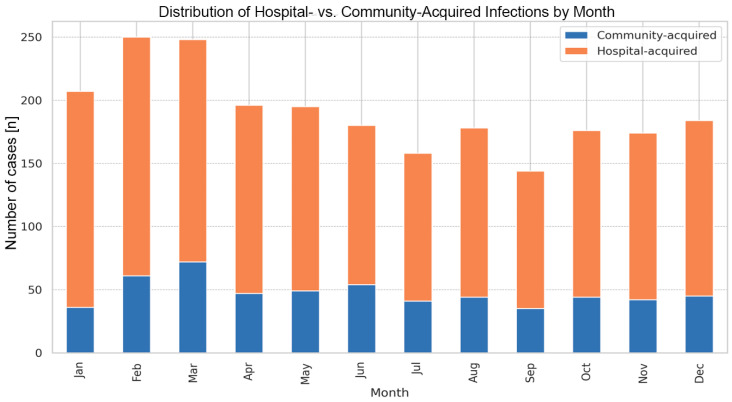
Occurrence of community- and hospital-acquired *Clostridioides difficile* infections among adult patients at the University Hospital in Kraków, monthly analysis (2016–2022).

The average time from admission to diagnosis for HA-CDI cases was 11 days (Table 1). Monthly analysis did not reveal statistically significant seasonal predispositions for HA-CDI versus CA-CDI. However, the highest HA-CDI/CA-CDI ratio was observed in January (4.75), and the lowest in June (2.33) (Figure 1).

Among the analyzed subpopulations, the geriatric unit had the highest CDI incidence (23.9/1000 patients). The proportion of HA-CDI was statistically significantly lowest in the ID Unit, at 59.6% (*p* < 0.001, Table 3). rCDI occurred in 317 patients (13.8% of CDI cases), ranging from 10.3% in 2019 to 15.3% in 2018, with similar rates across subpopulations. In total, the incidence of rCDI was 0.6 per 1000 admissions. No significant differences were found in rCDI rates across studied wards.

Antibiotic treatment was initiated in 93.4% of CDI cases. In the first episode, the most used regimen was therapy with vancomycin and metronidazole (41.9%), closely followed by oral vancomycin monotherapy (37.4%). Fidaxomicin was used infrequently, in only nine cases (0.4%) (Table 1). In cases not receiving CDI-specific antibiotic therapy, specific reasons were not documented; however, a notable subgroup included patients waiting for ELISA confirmation. Among re-infection cases, 96.1% received similar treatment regimens as during the initial episode, but with domination of therapy (vancomycin, metronidazole), which was used in 55.6% of cases, followed by vancomycin monotherapy (31.8%).

**Figure 2:**
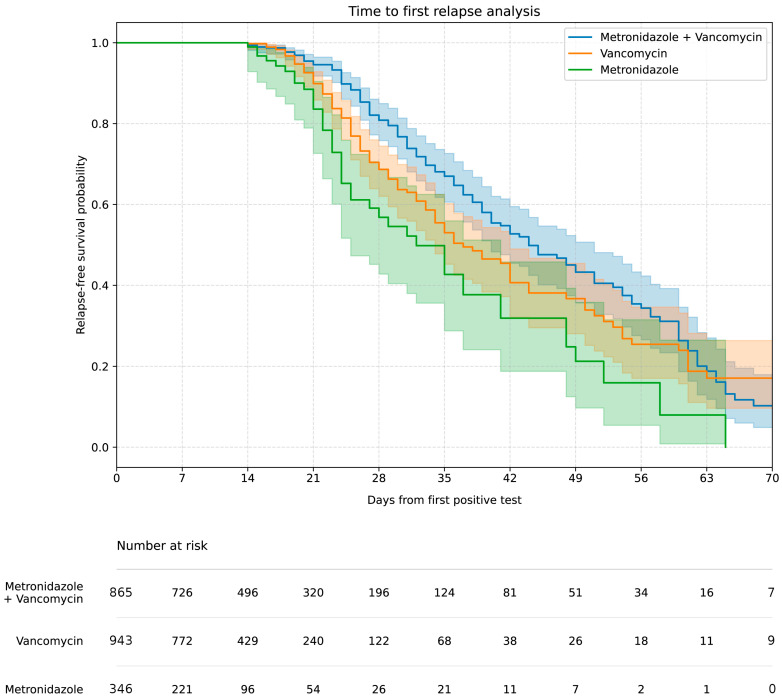
Kaplan–Meier curve of the relapse-free survival rate after successful treatment including treatment options.

The highest rCDI rate (approx. 17%) was associated with initial vancomycin, metronidazole therapy (concomitant or sequential therapy), compared to 12–13% for monotherapy (*p* < 0.01). The Kaplan–Meier curve (Figure 2) showed the highest 60-day relapse-free survival in the vancomycin, metronidazole therapy group and the lowest in the metronidazole monotherapy group.

Of all CDI patients, 73.7% were discharged, 3.6% were transferred for further treatment, and 22.5% died. The mean time from CDI test to death was 11.0 days (Table 1). Case fatality rates (CFRs) were highest among those initially treated with combination therapy (29%) and lowest among those treated with vancomycin monotherapy (17%).

After adjusting for age, initial vancomycin therapy was associated with a significantly lower risk of death compared to metronidazole (OR 0.73, 95% CI 0.56–0.95; *p* = 0.02), whereas therapy with vancomycin, metronidazole (concomitant or sequential therapy) predicted higher CFRs (OR 1.35, 95% CI 1.05–1.75; *p* = 0.02).

## 3. Discussion

During the COVID-19 pandemic, the overall incidence of CDI did not increase, and the risk of recurrent CDI (rCDI) decreased to approximately 11%. However, the proportion of HA-CDI tripled. On the other hand, the HA-CDI incidence in our hospital (3.1% between 2016 and 2022) was comparable to that reported by Gilboa et al. in Israel (3.4% between 2017 and 2023) [19]. The distribution of CDI cases—HA-CDI vs. CA-CDI—showed a typical 2:1 ratio, consistent with ECDC data and findings by Finn et al. [20]. In 2020, the HA-CDI/CA-CDI ratio surged to nearly 7:1, reflecting the impact of the early pandemic phase, as supported by our previous research on healthcare-associated infections and the spread of *Acinetobacter baumannii* ST600 [17,18]. On the other hand, these increases in CDI incidence rates align with previous reports indicating a higher incidence of CDI among patients with COVID-19. Proposed explanations include the severe course of COVID-19 during the early phase of the pandemic, widespread antibiotic use, and overlapping gastrointestinal symptoms in both infections [21,22,23]. It is estimated that around 72% of COVID-19 patients received broad-spectrum antibiotics—primarily respiratory quinolones—to prevent bacterial co-infections and superinfections [24]. In the studied hospital, antibiotic consumption nearly doubled in 2020 [16], which likely represents the main driver of the increase in HA-CDI observed in our study. During the COVID-19 pandemic, the infection prevention and control team developed and implemented hospital hygiene protocols, accompanied by training sessions on their appropriate use. However, it must be noted that at the peak of the pandemic, the number of cases was so high that many individuals with limited experience in the care of patients with infectious diseases—or limited clinical experience in general (e.g., physicians previously not working in hospital settings or medical students)—were recruited to support pandemic response efforts. This situation may have compromised adherence to hygiene protocols.

The CDI testing rate in our hospital was comparable to ECDC data, at approximately three tests per 100 hospitalizations. However, the proportion of positive results was higher than the EU average (14.5% vs. 10%). The diagnostic algorithm—initial screening for glutamate dehydrogenase (GDH) via enzyme immunoassay (EIA), followed by confirmation with EIA for toxins A/B—was consistent with ECDC surveillance methodology and ESCMID recommendations [9].

The highest CDI incidence rates were observed among patients in the geriatrics and ID units, particularly in the post-COVID-19 period. HA-CDI accounted for over 75% of cases, with the highest rates observed in the ID Unit, which is consistent with expectations. CDC data indicate that CDI incidence—regardless of origin—is more than twice as high in patients aged 75 years and older compared to younger populations [25]. Similarly, ECDC reports advanced age as one of the strongest risk factors for CDI in hospitalized patients across Europe [9].

Recurrent CDI occurred in 14.2% of the studied population. Vancomycin-based treatment was associated with a lower CFR compared to metronidazole, while vancomycin, metronidazole therapy showed the highest recurrence rate. Notably, findings by Julie Keating et al. suggest a lower (though not statistically significant) rCDI rate in patients treated with oral vancomycin compared to placebo, supporting our observations regarding vancomycin’s safety profile [26].

A systematic review by Finn et al. (2009–2019) reported a global rCDI rate ranging from 10% to 20%, with a median of 17.0% [20]. In our study, the overall rCDI rate was 14%, consistent with global estimates. Interestingly, during the COVID-19 pandemic, the rCDI rate declined to approximately 10%, despite a twofold increase in antibiotic consumption at the UHK [16]. Using the ECDC metric, the hospital incidence of rCDI in our setting (0.6 per 1000 discharges) was more than twice the EU average (0.24–0.27 between 2018 and 2020), underscoring the impact of antimicrobial stewardship on CDI epidemiology.

Recent IDSA/SHEA and ESCMID guidelines recommend fidaxomicin as the first-line therapy for both non-severe and severe initial CDI episodes because of its superior efficacy in preventing recurrence. Vancomycin remains an acceptable alternative, whereas metronidazole is no longer preferred for non-complicated disease. Furthermore, the 2021 EUCAST guidelines advise against combining intravenous metronidazole with oral vancomycin in CDI management [27]. For severe–complicated cases, IDSA/SHEA continue to endorse combination therapy with high-dose oral and/or rectal vancomycin together with intravenous metronidazole, while ESCMID additionally supports the use of fidaxomicin and tigecycline as part of combination regimens [28,29].

The last reassessments of metronidazole’s role in fulminant CDI have, however, challenged the evidence base for its removal from recommended therapeutic strategies. Pipitone et al. critically reviewed the observational studies underpinning recent ESCMID and IDSA/SHEA recommendations and emphasized substantial methodological limitations. Their meta-analysis found only a minimal and statistically non-significant difference in mortality between patients treated with vancomycin alone and those receiving combination therapy with intravenous metronidazole, alongside marked heterogeneity across studies. These findings suggest that the decision to include intravenous metronidazole—particularly in fulminant cases where impaired gastrointestinal absorption may reduce intraluminal drug availability—should remain individualized rather than being universally dismissed [30].

In Poland, access to fidaxomicin remains significantly limited due to its high acquisition cost, which places it among the most expensive antibiotics available on the market. Consequently, its use in routine clinical practice is very limited, which is also reflected in our results. Therefore, despite the comparable position of fidaxomicin and oral vancomycin in international treatment guidelines, the significant cost difference between these drugs (approximately EUR 830 vs. EUR 19) is a key factor influencing treatment decisions and favors the use of vancomycin in healthcare systems facing persistent financial constraints. By comparison, CDC data show that vancomycin also remains the most commonly used CDI treatment in the United States, although fidaxomicin accounted for 12% of prescriptions in 2022, while in our hospital, it was used in less than 1% [25].

In our study, conducted in a setting with limited access to fidaxomicin, the highest 60-day relapse-free survival rate was observed in patients treated with concomitant or sequential therapy with vancomycin, metronidazole. Notably, the higher case fatality rate (29%) and recurrence rate (17%) recorded in this group are most likely attributable to confounding by indication, as such therapy was preferentially reserved as a salvage strategy for patients with the most severe clinical presentations. Consequently, these outcomes should not be interpreted as evidence of inferior efficacy of combination therapy compared with monotherapy, but rather as a reflection of greater baseline disease severity in this patient population.

Bezlotoxumab is currently unavailable in Poland, further limiting therapeutic options for recurrence prevention. In contrast, in the United States, live biotherapeutic products—such as fecal microbiota, live-jslm (REBYOTA), and fecal microbiota spores, live-brpk (VOWST)—are recommended after antibiotic therapy to prevent second or subsequent rCDI episodes [31,32].

The in-hospital case fatality rate observed in our study (22.5%) was slightly higher than that reported by Cruz et al. (18.3%) [33], and notably higher than the 9% reported by Guh et al. in a large U.S. cohort [4]. These differences may reflect variations in patient demographics, disease severity, and access to advanced therapies such as fidaxomicin or bezlotoxumab.

CDI prevention is still challenging, as evidenced by recently published data from a CDI risk reduction study. The study included 39,046 participants in the pre-AI period and 40,515 participants in the post-AI period and showed that, unfortunately, even implementing an AI-assisted infection prevention package did not significantly reduce the incidence of CDI [34].

## 4. Materials and Methods

### Study Design and Study Population

This retrospective, observational, laboratory-based study was conducted at the UHK, the largest multi-specialist hospital in southern Poland, with a total capacity of 1280 beds, including 52 intensive care unit (ICU) beds. Between 2016 and 2022, a total of 528,887 patients were hospitalized at the UHK. From October 2020 to March 2022, dedicated COVID-19 units were established within the hospital. Based on the primary reason for hospitalization, the study population was stratified into four subgroups: patients admitted to (1) intensive care units (ICUs), (2) geriatric wards, (3) gastroenterology and infectious diseases wards (ID Unit), and (4) all other departments.

Inclusion criteria were as follows:

- Age ≥ 18 years;
- Hospitalization at the UHK between 2016 and 2022;
- Presence of symptoms suggestive of active CDI at admission or during hospitalization;
- An ordered for CDI diagnosis.

Information regarding patients’ sex, age, duration and location of hospitalization, diagnostic procedures for CDI, and administered antibiotic therapy was retrieved from electronic medical records. Detailed data on antibiotic prescriptions were obtained directly from the hospital pharmacy database. Vancomycin ± metronidazole therapy was defined as concomitant or sequential administration of vancomycin and metronidazole. No validation of the extracted data was performed, and the dataset may have contained erroneous or incomplete entries due to limitations in routine electronic medical records. The hospital did not maintain a registry of FMT, and therefore information on its use was unavailable.

A case of CDI was defined as a patient presenting with symptoms of variable severity and meeting diagnostic criteria based on the following algorithm. Initial screening for glutamate dehydrogenase (GDH) was performed using an enzyme immunoassay (EIA), followed by confirmation with an EIA for toxins A/B. CDI was diagnosed when

- Both GDH antigen and toxins A/B were detected using an immunoenzymatic assay (C.DIFF QUIK CHEK COMPLETE, TECHLAB, Blacksburg, VA, USA);
- An equivocal immunoenzymatic result was confirmed by an enzyme-linked immunosorbent assay (ELISA) (C.DIFFICILE TOX A/B II, TECHLAB, Blacksburg, VA, USA),

in accordance with the 2017 IDSA/SHEA Clinical Practice Guidelines [28]. Stool cultures for toxins and cytotoxicity neutralization assays were not performed at the study site.

CDI cases were classified according to the ECDC surveillance protocol (version 2.4) [35] as follows:

- Community-acquired CDI (CA-CDI): Symptom onset <48 h after admission and the *C. difficile*-positive stool specimen was collected on an outpatient basis or within >48 h after hospital admission in a person with no documented overnight stay in a healthcare facility in the preceding 12 weeks;
- Hospital-acquired CDI (HA-CDI): Symptom onset and specimen collection > 48 h after hospital admission;
- Recurrent CDI (rCDI): Recurrence of symptoms within 2–8 weeks following resolution of a previous episode, with or without treatment.

Ethical approval was waived by the Bioethics Committee of the Jagiellonian University (approval no. KBET 118.6120.42.2023, issued on 15 June 2023), in view of the retrospective nature of the study, all procedures being performed as part of routine care, and the analysis not including any participant-identifying data. All data analyzed during this study were anonymized prior to the analysis. As a result, no informed consent was required from the participants. This report adheres to the Standards for Quality Improvement Reporting Excellence (SQUIRE) guidelines.

## 5. Limitations

This study was limited by its retrospective analysis, which relied on the UHK IT systems used to maintain standard medical records. Therefore, data on the temporal relationships of antibiotics used may be subject to errors resulting from the limitations of the IT system as well as the way the system generates data. No formal validation of the extracted dataset was performed, and the absence of systematic chart review means that ICD-10-based case identification could not be independently verified.

Due to the manual linking of microbiological test results with hospitalization data, some data may have been lost, particularly in the context of treatment. To ensure reliable analysis, the data were standardized against similar reports prepared by the unit regarding antibiotic consumption.

## 6. Conclusions

Our study highlights the complex interplay between the COVID-19 pandemic and the epidemiology of *Clostridioides difficile* infections. While the overall CDI incidence remained stable, a marked increase in healthcare-associated cases and a shift in recurrence patterns were observed. The findings underscore the significant impact of antibiotic overuse, particularly during the early pandemic phase, on CDI dynamics. The highest CDI incidence rates were recorded in geriatric and infectious diseases units, reflecting the vulnerability of older adults and immunocompromised patients. Despite limited access to fidaxomicin and bezlotoxumab in Poland, vancomycin-based regimens demonstrated favorable outcomes, especially in terms of mortality reduction.

The elevated rCDI incidence compared to EU averages, alongside high in-hospital case fatality rates, points to the urgent need for improved antimicrobial stewardship, expanded access to advanced therapies, and enhanced diagnostic protocols.

In conclusion, optimizing CDI management in resource-limited settings requires a multifaceted approach, including rational antibiotic use, adherence to evidence-based treatment guidelines, and investment in novel therapeutic options.

## Figures and Tables

**Table 1:** Baseline characteristics, diagnostic pathways, treatment, and outcomes of patients with laboratory-confirmed CDI hospitalized in University Hospital in Kraków in 2016–2022.

Observed *Clostridioides difficile* Infection	Cases
Patients with CDI, n (%) Positive basic test ELISA confirmation Total	2341 (87.4%) 328 (12.6%) 2679 (100.0%)
CDI patients, gender (F), n (%)	1220 (52.1%)
CDI patients, age [years], median (IQR)	72.0 (61.0; 82.0)
HAI, n (%)	1665 (71.1%)
HAI cases, admission to result [days], Me (IQR)	11.0 days (6.0; 19.0)
Treatment, n (%) Vancomycin Metronidazole Vancomycin, metronidazole Fidaxomicin Total	876 (37.4%) 326 (13.9%) 982 (41.9%) 9 (0.4%) 2186 (93.4% of all CDI cases)
CDI patients not receiving CDI-specific antibiotic therapy, n (%)	
Death ELISA confirmation No data Total	29 (18.7%) 49 (31.6%) 77 (49.7%) 155 (6.6% of all CDI cases)
In-hospital outcome, n (%) Discharged home Transferred to another hospital ward Death	1726 (73.7%) 85 (3.6%) 527 (22.5%)
Result to death, median (IQR)	11.0 days (4.0; 26.0)
Re-infection, n (%)	333 (14.2%)
Re-infection group treatment, n (%) Vancomycin Metronidazole Vancomycin, metronidazole Fidaxomicin Total	106 (31.8%) 29 (8.7%) 185 (55.6%) 0 (0.0%) 320 (96.1% of all CDI cases)

Legend: CDI—*Clostridioides difficile* infection; F—females; HAI—hospital-acquired CDI; IQR—interquartile range; Me—median.

**Table 2:** Epidemiological summary, incidence, mode of acquisition, and recurrence of CDI of patients with laboratory-confirmed CDI hospitalized in University Hospital in Kraków in 2016–2022.

Year	Admissions	CDI Cases	Mode of Acquisition			rCDI Cases
			CAI Incidence *	HAI Incidence *	HAI/CAI Ratio	
2016	80,104	302	1.3	2.5	2.0	41 (13.6%)
2017	81,444	351	1.4	2.9	2.1	69 (19.7%)
2018	79,964	348	1.5	2.9	2.0	55 (15.8%)
2019	80,617	373	1.3	3.3	2.5	40 (10.7%)
2020	55,440	239	0.6	3.8	6.7	26 (10.9%)
2021	68,154	344	1.4	3.7	2.7	46 (13.4%)
2022	83,164	384	1.4	3.2	2.3	56 (14.6%)
Total	528,887	2341	1.3	3.1	2.5	333 (14.2%)

* Incidence per 1000 admissions. Legend: CAI—community-acquired infection; CDI—*Clostridioides difficile*; F—females; HAI—hospital-acquired infection; rCDI—recurrent CDI.

**Table 3:** *Clostridioides difficile* infections and antibiotic used in study population of patients hospitalized in University Hospital in Kraków, Poland, and admitted to intensive care units (ICUs), geriatric wards, gastroenterology and infectious diseases wards (ID Unit), and all other departments in 2016–2022.

Studied Subpopulations	ID Unit N = 270	ICU N = 94	Geriatrics N = 463	Others N = 1463	*p*-Value
Incidence *	19.5	13.1	23.9	3.0	<0.001
HAI n (%)	161 (59.6%)	87 (92.6%)	396 (85.5%)	1076 (73.6%)	<0.001
Re-infection n (%)	37 (13.7%)	11 (11.7%)	61 (13.2%)	208 (14.2%)	0.871
Vancomycin n (%)	211 (78.2%)	84 (89.4%)	333 (71.9%)	992 (67.8%)	<0.001
Metronidazole n (%)	121 (44.8%)	71 (75.5%)	266 (57.5%)	690 (47.2%)	<0.001
Fidaxomicin	0 (0.0%)	0 (0.0%)	0 (0.0%)	12 (0.8%)	0.078

* Incidence per 1000 admissions. Legend: HAI—hospital-acquired infection; ICU—intensive care unit; ID Unit—gastroenterology and infectious diseases wards.

## Data Availability

The datasets analyzed during the current study are not publicly available due to being internal hospital documents, but are available from the corresponding author on reasonable request.
